# The dear enemy effect drives conspecific aggressiveness in an Azteca-Cecropia system

**DOI:** 10.1038/s41598-021-85070-3

**Published:** 2021-03-17

**Authors:** Gabriela Zorzal, Flávio Camarota, Marcondes Dias, Diogo M. Vidal, Eraldo Lima, Aline Fregonezi, Ricardo I. Campos

**Affiliations:** 1 Programa de Pós-Graduação Em Ecologia, Departamento de Biologia Geral, Universidade Federal de Viçosa, Viçosa, Minas Gerais 36570900 Brazil; 2 Departamento de Entomologia, Universidade Federal de Viçosa, Viçosa, Minas Gerais Brazil; 3 Departamento de Química, Instituto de Ciências Exatas, Universidade Federal de Minas Gerais, Belo Horizonte, Minas Gerais Brazil

**Keywords:** Behavioural ecology, Community ecology

## Abstract

Territoriality is costly, and the accurate identification of intruders and the decision to perform aggressive responses are key behavioral traits in social animals. We studied aggression among individuals belonging to close and distant nests of the plant-ant *Azteca muelleri*, which lives in stems of the pioneer tree *Cecropia glaziovii*. More specifically, we aim to investigate if the DE (dear-enemy effect—less aggression towards neighbors than strangers) or NN (nasty-neighbor effect—less aggression to strangers than neighbors) effects or even none of them apply for this iconic Azteca-Cecropia system. We further checked if ant aggression towards conspecifics is related to cuticular hydrocarbon profiles (CHCs), which provide chemical cues for nestmate recognition. Therefore, we sampled 46 nests of *A*.* muelleri *in three Brazilian Atlantic forest fragments and performed behavioral trials within and between sites. Consistently with the DE effect, we found higher aggression levels in ‘between sites’ versus ‘within sites’ treatments as well as a positive effect of spatial distance on ant aggressiveness. We found no effect of the overall dissimilarities on CHC blend on ant aggressiveness, but of one CHC class, the methylated alkanes. Overall, we provide key insights on nest-mate recognition in obligatory ant-plant mutualisms.

## Introduction

Territorial defense behavior is a remarkable feature of animal societies^1,2^. Territoriality is costly since it implies the use of defensive forces. The accurate identification of intruders and the decision to perform aggressive responses are key behavioral traits in social animals^3^. The aggression levels to intruders have received substantial research attention, and generally, two opposite outcomes are expected^4^. Firstly, when distant invaders are potentially more dangerous than closer ones regarding resource-threatening, territorial animals will respond less aggressively to neighbors than strangers (named as “dear enemy” effect—DE)^5,6^. Distant invaders can be more threatening when: (1) their colonies boundaries are not well-know, (2) their intrusions are less predictable in space and time, and (3) when they are looking for new territories^7^. On the other hand, if resource competition is stronger between closer conspecifics, higher aggressions should be displayed to neighbors than strangers (known as “nasty neighbor” effect—NN)^8^. The NN effect would be more likely when transient strangers are smaller or when resource levels are fluctuating, encouraging usurpation of available resources by neighbors^4^. Notably, the optimal level of territorial aggression is highly context-dependent, with the interaction outcome influenced by the life-histories of the involved organisms^6,9^.

Among social animals, ants represent an outstanding example of social organization, which may be a key factor explaining their ecological dominance in terrestrial ecosystems^10–12^. Importantly, ants might be aggressive and are especially efficient in chemically recognizing nestmates from non-nestmates^13,14^. Therefore, ants represent appropriate biological models to investigate neighbor-stranger discrimination and aggression. However, like it is also true for other animals, there are mixed pieces of evidence, with some ant behavioral studies pointing to a DE^15–17^ and others to NN effects^18–20^. Besides, there are even some cases where no clear signs of neighbor-stranger discrimination occur^21,22^.

Despite the increasing number of studies focusing on ant nestmate recognition and aggressiveness^23–25^, this literature is mainly focused on ground ant species^26^, and studies on arboreal ants are rare. However, arboreal ants are often involved in mutualistic interactions with their host-plants, and aggressiveness often play a key role in shaping these ant-plant relationships^27^. For example, to our knowledge, while there are studies involving neighboring colonies on ant-plants^28–30^, no published paper has specifically studied nestmate recognition for obligatory mutual relationships between ants and plants. However, it is well known that, in exchange for food and shelter, plant-ants present a highly aggressive behavior against their host plant’s natural enemies^31^. Both the NN and the DE are possible regarding neighbor-stranger aggression among plant-ants. The NN would more likely occur when the colonies’ borders are not well delimited, and potential invasions are harder to predict^4,7^. Additionally, when local nesting site availability is low, neighbors are competing for the few available nesting sites left, generating the NN. The DE would be more likely if the different colonies have well-defined borders^7^, generating high temporal and spatial predictability of the colony boundaries. Here, we investigated these two possible outcomes, NN or DE, and aim to understand if chemical recognition promotes intercolonial aggression.

Ants perform nestmate recognition mainly based on chemical cues, mostly involving cuticular hydrocarbon profiles (also called CHCs)^32,33^. Each colony has its own chemical identity (“colony odors”), which forms an odor template used to discriminate between nestmates and invaders^34–36^. Recognition cues are thought to be compared to a template that resides in the peripheral and central nervous system^37^. Any incompatibility between intruders and template odors may result in aggression^38–40^. Colony recognition might be particularly important in obligatory mutualisms, where the loss of the territory, i.e., the host plant, implies the death of the whole colony^31,41^. Despite that, there are no empirical studies investigating neighbor-stranger discrimination and aggression in the heavily studied obligatory ant-plant systems.

Based on this, we studied recognition and aggression among individuals belonging to close and distant nests of the plant-ant *Azteca muelleri*, which lives in hollow stems of the pioneer tree *Cecropia glaziovii*. Like other mutualistic *Azteca* species^41,42^, *A*.* muelleri* is strongly aggressive towards herbivores and effectively protects its host plant^43^. More specifically, we aim to investigate if the DE or NN effects or even none of them apply to this Azteca-Cecropia system. To take a step forward, we further checked how ant aggression towards conspecifics was related to differences in CHC profiles between ant colonies. More specifically, we designed the study to answer the following questions: (a) Is there an effect of spatial distance on ant aggressiveness? (b) Is there an effect of chemical distances on ant aggressiveness?

## Methods

### Study area

**Figure 1:**
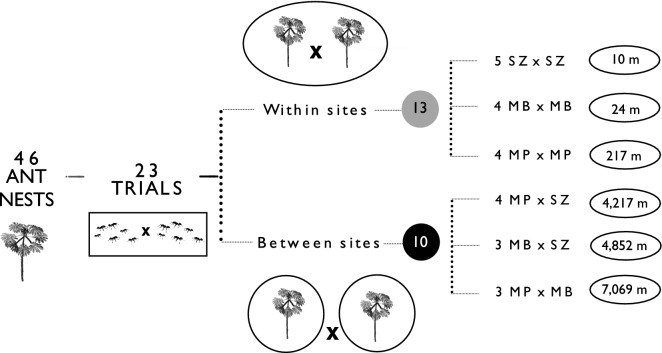
Schematic representation of behavioral trials showing the number of ant nests (trees) and trials within and between the three sampling sites. The number within circles represent the average spatial distance between ant nests (trees) that were engaged in behavioral trials from the six possible combinations of sites. Within sites—MP versus MP; SZ versus SZ; MB versus MB and between sites: MP versus MB; MP versus SZ; MB versus SZ. SZ—“Seu zé”; MB—“Mata da Biologia” and MP—“Mata do paraíso”.

We sampled *A*.* muelleri* colonies in three Atlantic Forest fragments located in Viçosa town (20°48′ 07′′ S, 42°51′ 31′′ W), state of Minas Gerais, Southeastern Brazil: “Mata do Paraíso” (MP), “Mata da Biologia” (MB) and “Mata do Seu Zé” (SZ). The region has a subtropical climate, with annual precipitation of 1300 to 1400 mm and an average temperature of 19 °C^44^. All sites are equally comprised of secondary Seasonal Atlantic Forest vegetation^45^, but they have different sizes and regeneration times. The MP is a research station from the Federal University of Viçosa (UFV), with an area of 195 ha, and ~ 60 years of regeneration process following coffee plantation. MB is located within the university campus, has an area of 75 ha, and more than 90 years of regeneration, following cattle-grazed pastureland and coffee plantation. The SZ is a private area, with ~ 20 ha, and its vegetation has approximately ten years of regeneration after cattle pasturelands. We calculated the distance between sites through the online geographic calculator of the “Instituto Nacional de Pesquisas Espaciais” (INPE), where the distance in meters between the sites and between the plants in each site was estimated using GPS data (see in Fig. 1). All parts of this work comply with the current research laws of Brazil. Besides, we had all the specific permits from the SZ owner and the UFV environmental managers to sampling in our three sites.

### Biological system

*Cecropia glaziovii* Snethl. (Cecropiaceae), is a fast-growing tree usually found in Forest regeneration fragments, reaching up to 20 m in height and restricted to an altitudinal range between 600 and 1500 m^46^. *Cecropia glaziovii* is usually involved in mutualistic associations with the ant *Azteca muelleri *(Dolichoderinae)^47,48^, an aggressive ant species that is an effective protector of its host plant against herbivores and other ants^49^. Indeed, there is often fierce intra- and interspecific competition between *Azteca* colonies for nesting sites within their *Cecropia* hosts^50–52^^.^ The density of Azteca colonies inhabiting Cecropias is strictly related to the density of their plant host^41,52^. While we have no data concerning nest density and dispersal of *A*.* muelleri* in our focal region, prior studies on other *Azteca* species showed that queens could disperse to long distances^53,54^. While *A*.* muelleri* can colonize different species of *Cecropia*^55^, we found only *C*.* glaziovii* as a host plant of *A*.* muelleri* in our three sampling areas.

### Ant-plant sampling

We sampled 46 *A*.* muelleri* nests located inside 46 *C*.* glaziovii* individuals’ plants, being 14 on MP, 12 in MB, and 20 in SZ, between February–April 2017 and August–November 2018. In this way, we could carry out experiments with plants from all locations in different seasons (dry and rainy). On each plant, we scanned *A*.* muelleri*’s workers’ presence on the plant surface by beating the trunk. If there were active workers, then we measured the plant height. As prior studies have found a strong relationship between the host-plant size and colony size and age for ant-plants^56,57^, we further used tree height as a proxy of colony size and age. After detecting active workers, we cut the plant and collected the alive queen and around 50 ant workers per plant and stored it in perforated plastic pots together with a small piece of the upper part of each tree trunk. We considered a colony unit all the ants within a single plant, where we found only one queen. Plants with no queens or with two or more queens were not considered. Therefore, our sampling size was constrained by the availability of whole individual trees in the studied area (in total, 125 trees were collected, including those used for pilot sampling and final tests). The plastic pots were stored in a breeding room with controlled humidity and temperature (45% and 25 °C, respectively) at the “Laboratório de Ecologia de Formigas” from “Universidade Federal de Viçosa”. After the trials (which will be detailed in the next section), we collected ant individuals for each colony, which had their identity assessed morphologically by ant taxonomists. Alternatively, we also sequenced the mitochondrial gene partial region that encodes cytochrome oxidase subunit I (COI). We used the COI data as a ‘genetic barcode’ to confirm that ants collected from our three sites belong to the same species, i.e. *Azteca muelleri* (see details in Online Resource S1, Fig. S1).

### Behavioral trials

For the ant behavioral tests, we performed 23 trials using 46 nests. Each colony was engaged in only one behavioral trial. We performed one trial per nest pair since even collecting a few individuals could bring more stress to the whole colony (which was already under stress in laboratory conditions). The trials were divided into two distinct groups. For the first group (hereafter ‘within sites’), we ran 13 trials and used workers from different colonies but the same site. For the second group (hereafter ‘between sites’), we ran ten trials and also used workers from different colonies, but now from different sites (Fig. 1). For each trial, we placed ten *A*.* muelleri *individuals from two colonies (five individuals per colony) in a 25 cm^2^ plastic Gerbox arena and observed their interactions for 5 min. We spread odorless talc powder only in the Gerbox upper border to prevent ants from escaping the arena. Before each behavioral trial, we divided our arena into two parts using a 5 cm plastic ruler barrier, and after placing the ants in the arena (each colony on one side of the barrier), we waited for 5 min for ant acclimation. After that, we removed the plastic rule and let the ants interact freely inside the arena for 5 min. *Azteca* ants belonging to the same species can be highly variable morphologically^57^ as it is common for ants in general^58,59^. Noticeable differences in size and color can be related to the colony's maturity stage and aspects of the host-plant^57^. Additionally, before any behavioral trial, we carefully observed the colony to obtain cues over their morphological differences. Therefore, we were able to distinguish the ant individuals from the two different colonies. Finally, we did the last assessment after performing the trials by assuring that there were no aggressive interactions between the ants considered from the 'same colony,' as it is well known that ants belonging to the same colony do not attack each other. During these 5 min, we classified all the behaviors of each pair of interacting ants following a modified version of the ‘protocol of aggressiveness’ proposed by Holway et al. (1998)^60^ and adapted by Giraud et al. (2002)^61^. Thus, every time one ant from a different nest approaches each other, we classified the behavioral interactions between then into six levels on a scale from 0 to 5 as follows: 0—ignore, has no physical contact and shows no interest; 1—antennation, repeated antenna taps on the other ant body; 2—avoidance, retract to the opposite direction after contact; 3—dorsal flexion of the gaster and mandible opening; 4—aggression, bites or pulls the head, legs or other parts of the body and 5—fight, prolonged aggression, locking the jaw in one body part of the other ant, not releasing until the end of the trial. Based on this scale, we consider the scores 0–2 as “non-aggressive” and 3–5 as “aggressive” behaviors. After that, we calculated a behavioral index by summing the number of times (frequency) that each behavior was scored and dividing this number by the total number of interactions displayed within 5 min. For example, in a trial where we observed two interactions scored as level 2, and one as level 3, our behavioral index would be 2.5 = (2 * 2 + 1 * 3)/3.

### Chemical analyses

After the aggression tests, we weighted the ants and stored them at − 22 °C until the extraction of the cuticular hydrocarbons (CHCs) by hexane. The extraction was performed by immersing five workers per nest in 50 μl of hexane for 2 min, followed by the removal of the supernatant. On each sample, we added 50 µL^−1^ of *E*, *E*-Farnesol (Sigma-Aldrich, St. Louis, MO, USA), with a concentration of 50 ng µL^−1^ to the extracts as an internal standard^62^. This procedure was repeated three times in each nest in order to obtain triplicates of samples from each colony. For that, ants of similar weight were placed in each of the three samples. CHCs were quantified by GC-FID (Shimadzu GC-17A equipped with a Restek RTX-5 capillary column: 30 m × 0.25 mm × 0.25 µm), with a temperature program starting at 100 °C (1 min), with a maximum temperature of 280 °C (maintained for 10 min), after a heating ramp of 10 °C per minute^62^. Injector and FID temperatures were set at 250 °C. Samples were injected (1 µl) on splitless mode. Helium was used as a carrier gas, flowing at 1 ml per minute. Quantification data was used to measure the difference in hydrocarbon profiles between *A*.* muelleri* colonies. To avoid potential false-positive errors from GC-FID, we did not consider extracted compounds with a concentration lower than 1 ng µL^−1^. We also took out from our statistical analysis the chemical compounds with abnormally high standard deviation (i.e., greater than the mean) calculated from the triplicates of the same ant colony in order to avoid imprecise quantification. It is important to state that after performing these two compound exclusion methods above cited, we only discarded 7.15% (1467 out of 20,529 ng µL^−1^) of the total concentration of all compounds (9 out of 26) detected from our 46 ant nests.

Qualitative analysis was performed by GC–MS analyses (Shimadzu QP-2010 Plus equipped with a Restek RTX-5 capillary column: 30 m × 0.25 mm × 0.25 µm). Temperature and flow conditions were identical to the described for GC-FID analyses. Structural elucidation was performed based on retention indexes^63^, mass spectra interpretation, and NIST library. Retention indices (RIs) were determined using an *n*-alkane standard mixture (C_7_–C_40,_ Supelco, Bellefonte, PA, USA). Retention indices and mass spectra were used to compare and identify CHCs already described in the literature^63–65^.

Double bond positions of unsaturated compounds were determined based on the analysis of mass spectra obtained after the derivatization of natural extracts with DMDS^66^. The position of methyl groups on branched hydrocarbons was determined based on retention index patterns and the relative intensity of fragments on MS spectra^67^.

### Statistical analyses

To investigate the effect of spatial distance on ant aggressiveness, we used a general linear model (GLM), with spatial distance as the explanatory variable and the ant aggression index as the response variable. To test if ants presented higher aggression levels in ‘between sites’ versus ‘within sites’, we carried out a factorial GLM similar to a one-way ANOVA. For this, we used the site pairs (all the six possible combinations: within sites—MP vs. MP; SZ vs. SZ; MB vs. MB and between sites—MP vs. MB; MP vs. SZ; MB vs. SZ) as an explanatory variable (factor) and the aggression index as a response variable. Tukey HSD pairwise comparisons were performed after running the factorial GLM. After performing residual analyses, we discover that the models mentioned above followed Gaussian error distributions.

To test for the possible effect of chemical distances on ant aggressiveness, we first calculated the chemical distance between ant nests using two approaches. First, we calculated the Bray–Curtis index of dissimilarity among the overall chemical profiles (the concentration of all hydrocarbon compounds securely detected by IGC) between each pair of ant nests placed in behavioral trials (n = 23). Second, we calculated the same Bray–Curtis index of dissimilarity but now using separately the compounds belonging to the three most common classes of CHC as follows: linear alkenes, linear alkanes, and methylated alkanes. After that, we performed four GLM models using chemical distance (using the overall, linear alkenes, linear alkanes, and methylated alkanes CHCs separately) as the explanatory variables and ant aggression index as the response variable. Again, all the models described above followed Gaussian error distributions.

We used the R software^68^ for all statistical analyses and performed residual analyses by checking error distributions suitability for all models.

## Results

### Behavioral tests

**Figure 2:**
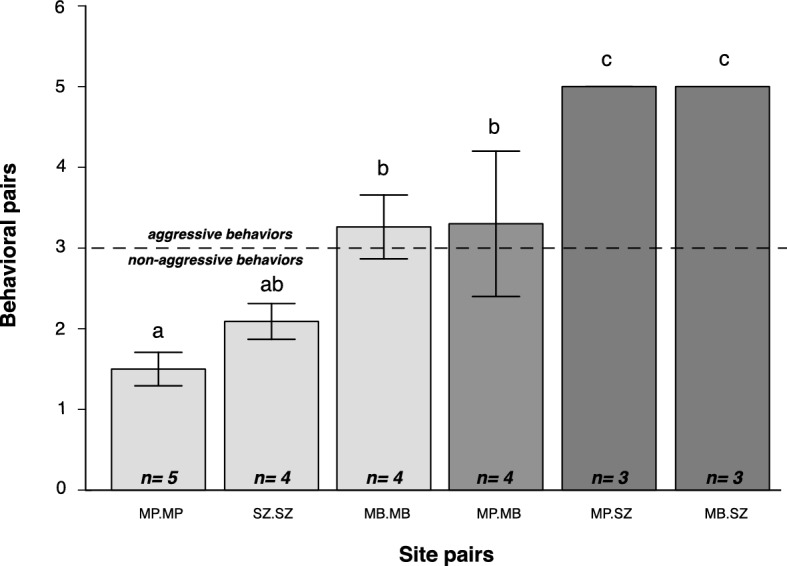
Mean (± SE) ant behavioral index per pair of sites comparing the six possible combinations of sites. Within sites—MP versus MP; SZ versus SZ; MB versus MB and between sites: MP versus MB; MP versus SZ; MB versus SZ. SZ—“Seu zé”; MB—“Mata da Biologia” and MP—“Mata do paraíso. Inside each bar is the number of trials within and between the three sampling sites.

We found that across the 23 pairwise behavioral tests, considering “within-sites” encounters, most interactions were non-aggressive (54.05%), while 45.9% were aggressive (Table 1). On the other hand, for the “between-sites” encounters, almost all interactions (97.5%) were aggressive (Table 1). Concordantly, when we compared the aggression index between the site pairs, we found that ant aggression was significantly higher in “between-sites” than “within-sites” trials (Fig. 2, F_5,17_ = 15.907, *P* < 0.001) (Fig. 2). Despite the higher aggression in “between-sites” trials, the comparisons between MB versus MB and MP versus MB did not differ from each other (Fig. 2).

**Table 1:** Outcomes from behavioral trails performed with ten *A. muelleri* ant individuals (five from each colony) placed in a plastic arena for 5 min.

Behavior	Frequency “within sites”				Frequency “between sites”				T. freq/behav
	MP.MP	MB.MB	SZ.SZ	% total	MP.MB	MP.SZ	MB.SZ	% total	
Ignore	2	0	2	5.4	0	0	0	0	4
Antennation	7	1	10	24.3	1	0	0	2.5	19
Avoidance	7	6	5	24.3	0	0	0	0	18
Gaster dorsal flexion/mandible opening	3	4	9	21.6	1	0	0	2.5	17
Aggression	0	4	5	12.2	1	0	0	2.5	10
Fight	0	4	5	12.2	7	20	10	92.5	46
T. Freq./pair of sites	19	19	36	100	10	20	10	100	114

The table shows the frequency (number of times) and the percentage (% total) that each behavior was scored in each trial where the ants (pair of nests) belonged to the same site (“within sites”) or different sites (“between sites”). Total number of behaviors from all trials (T. freq./behav.). Total number of behaviors from each pair of sites (T. Freq./pair of sites). SZ—“Seu zé”; MB—“Mata da Biologia” and MP—“Mata do paraíso”.

We ruled out ant’s weight and plant’s height as having a significant influence on ant aggressiveness, as we failed to find significant relationships (ant weight: *F*_1,21_ = 0.301, *P* = 0.59; plant height: *F*_1,21_ = 0.9472, *P* = 0.33). Therefore, these features of the colony structure did not explain the aggressive behavior between ants.

### Aggression versus spatial distance

**Figure 3:**
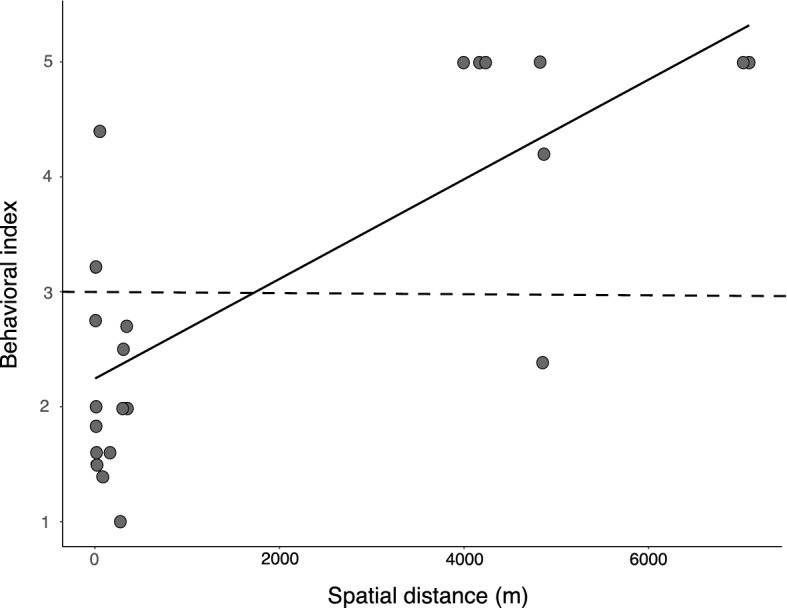
Relationship between the spatial distance and the ants’ behavioral index. Each point represents a pair of ant nests (five individuals per nest submitted to behavioral trials, N = 23). The solid line represents the linear regression fit to the data. The doted horizontal line indicates the threshold between non-aggressive (bellow the line) and aggressive (above de line) behaviors.

We found a positive influence of spatial distance on ant aggressiveness (Fig. 3, F_1,21_ = 30.098, *P* < 0.001) (Fig. 3). These results indicate a DE effect in our system once the aggressive behavior increase with the distance between nests.

### Aggression versus chemical distance

**Figure 4:**
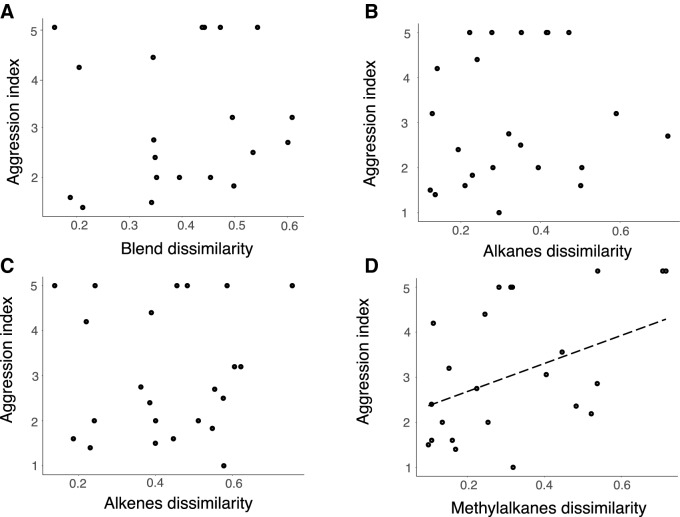
Relationship between ants’ CHC profile dissimilarity and the spatial distance between nests. (**A**) chemical dissimilarity using total CHC blend; (**B**) chemical dissimilarity using Alkanes only; (**C**) chemical dissimilarity using Alkenes only; (**D**) chemical dissimilarity using Methyl alkanes only. The solid line represents the linear regression fit to the data. Each point represents a pair of ant nests (the same ones submitted to behavioral trials, N = 23).

We detected a total of 26 CHCs in the *A*.* muelleri* chemical profile. Among these compounds, we observed three main classes: linear alkenes, linear alkanes, and methylated alkanes (Online Resource Table S1). After the compound selection analysis (see “Methods” section), a total of 15 CHCs were considered for the ant aggression analysis. There was no relationship between ant aggression and the overall chemical dissimilarity (Fig. 4A, F_1,18_ = 0.556, *P* = 0.46) (Fig. 4). When looking specifically for each of the three most important CHC classes, we found a positive relationship between methylated alkanes dissimilarity and the ant aggressiveness (Fig. 4D, F_1,21_ = 4.391, *P* = 0.048), whereas linear alkanes and alkenes were not significantly related with aggressiveness in our tests (Fig. 4B, F_1,21_ = 0.428, *P* = 0.52; Fig. 4C, F_1,21_ = 0.076, *P* = 0.78) (Fig. 4).

## Discussion

This study investigated neighbor–stranger aggressiveness in plant-ants, and by using an experimental approach, we found support for the “dear enemy” rather than “nasty neighbor” effects. Thus, we found that *A*.* muelleri *ants were more aggressive towards colonies located far away from their nests when compared to closer ones. We also checked for the importance of chemical recognition in leading the DE effect. We found no effect of the overall chemical blend but a significantly positive effect of one CHC class dissimilarity, the methylated alkanes. Our findings bring new light into the understanding of aggressive territorial defense in ants, and more specifically, in intricated ant-plant mutualisms^31,57,69^.

The differences in aggressive responses from neighbors or strangers are highly context-dependent, and factors such as resource predictability and colony delimitation can be essential to define the direction of the aggressive behaviors^70,71^. In ant-plant systems involving obligatory mutualisms, the ants rely on their host plants as shelter, and therefore there should be much competition for available nesting sites, i.e., whole plants^50–52^. After the colonization stage, the colonies of *A*.* muelleri* are well delimited, consisting of a single tree individual^7^. Thus, the borders between colonies are clear, which is an essential factor for the DE effect^19^. Well defined borders allow for a higher spatial and temporal predictability of the movement between nearby colonies^4^. More specifically, each neighboring colony of *A*.* muelleri* have already a delimited nest (one *C*.* glaziovii *tree) in their possession and so far poses less threat to another, especially because their status is generally known and they have less to gain from conflict. On the other hand, *A*.* muelleri *individuals coming from distant areas could be then recognized as bigger treats by the simple fact of being strangers.

The DE effect is widespread in ant communities^7,72^, and here we provide evidence that this effect is also important in obligatory ant-plant interactions. The main reason is that neighboring ant colonies already have an established resource (i.e., the whole tree), which is often restricted^18,73^. Moreover, in an obligatory ant-plant system, the host plant offers most of the necessary resources for the colony development and maintenance, and being dislodged from a tree means almost certainty colony death^74–76^. Thus, the host tree in an ant-plant mutualistic system might be ecologically interpreted as a typical example of an absolute territory^77–79^. As aggressive behavior is highly energy costly, the mutualistic ants might be able to recognize the enemies which present at the same time the highest threats for both the ants and the plants. As mutualistic ants normally present a high foraging activity on the tree and in its vicinities, they would have an increased probability of recognizing strangers as a greater threat than neighbors, ultimately increasing the DE effect.

The description of territorial defense behavior is much more common for vertebrates than other groups^6,80,81^. However, there is an increasing number of studies involving invertebrates, especially social organisms, such as ants^16,82^. Vertebrates can use distinct tactics for enemy recognition, including visual, olfactory, and vocal cues^6^. For ants, aggressiveness towards enemies is based primarily on chemical compounds^33,83,84^. Each ant colony has its unique odor composed of cuticular hydrocarbon compounds (CHCs), which form a chemical template that can guide the ant's behavior^85,86^. If intruder ants present an odor that does not fit the colony's chemical template, the resident ants often show an aggressive response^38,87^. However, our study could not find strong evidence that the entire blend of CHCs cuticle profiles is involved in eliciting aggression in *Azteca* ants living in our focal *Cecropia* trees. We have two non-exclusive lines of arguments to explain this, which will be presented in the next paragraph.

Firstly, insect cuticular lipids typically contain more than CHCs (e.g., fatty acids and esters), and these other chemical compounds may also have a role on nestmate recognition^83,88–90^. For example, for other ant species, like leaf-cutters^89–91^ and cuckoo ants^88^, the fatty acids are more important in nestmate recognition and aggressive behavior than CHCs. It could also be important for ants foraging on trees as the plant environment, i.e., its surface, contains fatty acids, which may be eaten by ants influencing cuticle chemical formation. Secondly, when analyzing the entire hydrocarbon template, we might be dealing with multiple functions at the same time that could be different from aggressiveness (e.g., mating attraction, food, and nest location)^92–95^. Specific hydrocarbons classes such as methyl-alkanes and alkenes have been related to aggressive behavior or conspecific recognition^39,40,96^, while alkanes can function as cuticle lubrication or even act as chemical barriers against microbes^97^. Here, we found evidence for the role of the methylated alkanes in guiding ant’s aggression, supporting the findings of other studies showing an important role for methyl-branched alkanes in nestmate recognition^95,98,99^. However, as the relationship between methyl-alkanes dissimilarity and ant aggression was not strong, these results should be interpreted with care, and potentially other factors might be of greater importance than CHC in eliciting aggression in *A*.* mull*eri colonies.

## Conclusion

Despite the “nasty neighbor” effect might occur more frequently in social insects^19,20,100^, we find here that the plant-ant *A*.* muelleri* is more aggressive to strangers than to neighbors, following the “dear enemy” effect^101^. We finally suggest that the DE effect might be related to mutualistic strength between partners^102,103^. Surprisingly, there is still a dearth of studies investigating territorial defense behavior in obligatory mutualistic systems. Therefore, we suggest that future studies directly investigate the relationship between neighbor-stranger conspecific aggression and mutualistic protection effectiveness.

## Supplementary information

Supplementary information.

## Data Availability

The authors intend to deposit the dataset at Dryad after the manuscript acceptance.
