# Supplementary material for: The dear enemy effect drives conspecific aggressiveness in an Azteca-Cecropia system
Source: Sci Rep. 2021 Mar 17;11:6158. doi: 10.1038/s41598-021-85070-3 (PMC7970830; doi:10.1038/s41598-021-85070-3)

**The dear enemy effect drives conspecific aggressiveness in an *Azteca-Cecropia* system**

Gabriela Zorzal, Flávio Camarota, Marcondes Dias, Diogo M. Vidal, Eraldo Lima, Aline Fregonezi & Ricardo I. Campos

**Supplementary Information**

**S1. Molecular analyses from cytochrome oxidase subunit I (COI)**

Here, we provide the first molecular barcoding for *Azteca mulleri.* For this, we choose eight individuals from eight different colonies distributed across the sampled areas (one colony from MB, three colonies from MSZ, and four colonies from MP). The abdomens of ant individuals were preserved in 96% ethanol and used for CTBA-based DNA extraction, adapted from Doyle & Doyle (1987). The mitochondrial gene partial region encoding the cytochrome oxidase subunit I (COI) was amplified by the PCR method with HCO1298 and LCO1490 primers, (described by Folmer *et al*. 1994), under 50˚C of annealing temperature. Amplified fragments were confirmed by 1% agarose gel electrophoresis and purified enzymatically (ExoI and SAP). After checking the chromatogram's quality, each individual's consensus sequence was determined by both strands with Geneious software v. 8.1.8 (http://www.geneious.com, Kearse et al., 2012). The obtained sequences were deposited at Genbank database under the accession numbers MT320882 - MT320889. After trimming sequences due to low-quality signals at the ends, the resulted alignment presented 531 base pairs with only one substitution and no parsimoniously informative sites. No stop codons or gaps were found. A BLAST comparison yielded a 99% coverage to a COI region of *Azteca nigricans*individuals from South America (Colombia) and *Azteca* sp. individuals from South America (Ecuador) and Central America (Panama), with E values ranging among 4e-147 - 2e-133 (number of expected hits of similar quality score that could be found just by chance). All samples showed about 82% identity. Unfortunately, there are no records for *Azteca muelleri*at the Genbank database to confirm our identification. Thus, we choose representative individuals of *Azteca* species available at the Genbank database to construct a Neighbour Joining tree (NJ), totaling 52 sequences and 204 parsimony informative sites. We aimed to make sure that our sequences truly belong to the same species. Since our sequences were smaller than some of those available at Genbank, we used 531 base pairs homologous to our alignment for the NJ tree construction. The species *Linepithema humile* and *Papyrus nitidus* were used as outgroups. The Kimura 2-parameter (K2P) distance metric with a gamma shape of 0.53, calculated by JModeltest 2.1.10 (Darriba et al., 2012), was employed for sequence comparisons (Kimura, 1980). Genetic distances, initial Neighbor-joining clustering, and confidence in estimated NJ tree topologies relationships were evaluated by bootstrap analysis with 1,000 replicates with MEGA version 10 (Kumar *et al*., 2018). The consensus NJ tree of COI sequences indicated that our samples formed a unique clade with a 100% bootstrap value (Figure S1). Since there were low support values related to the main clusters, it was not possible to infer the precise relationship of *Azteca muelleri* with other species of the genus. Despite the overall low bootstrap values obtained in the NJ tree, the degree of genetic variation found was sufficient to support *Azteca* species delimitation. Low support values shown in the NJ tree may reflect the few base pairs of the data set. Also, the effect of taxon subsampling could influence the support values of intra-species relationships (Nabhan & Sarkar, 2012; Hamilton *et al*., 2014). The *Azteca* genus samples at Genbank are uneven, with a major representation of individuals from Central America and very little South American species sampling, especially from the eastern South America region. Nevertheless, we aimed to confirm if our samples comprised a single species, and based on the NJ tree, they form a single evolutionary lineage.

**References**

Darriba D, Taboada GL, Doallo R, Posada D (2012) jModelTest 2: more models, new heuristics and parallel computing. Nat Methods 9:772.

Doyle JJ, Doyle JL (1987) A rapid DNA isolation procedure for small quantities of fresh leaf tissue. Phytochemical Bullettin 19:11-15.

Folmer O, Black M, Hoeh W et al (1994) DNA primers for amplification of mitochondrial cytochrome oxidase subunit I from diverse metazoan invertebrates. Molecular Marine Biology and Biotechnology 3: 294-299.

Hamilton CA, Hendrixson BE, Brewer MS, Bond JE (2014) An evaluation of sampling effects on multiple DNA barcoding methods leads to an integrative approach for delimiting species: a case study of the North American tarantula genus Aphonopelma (Araneae, Mygalomorphae, Theraphosidae). Molecular Phylogenetics and Evolution 71:79-93.

Kearse M, Moir R, Wilson A et al (2012) Geneious Basic: an integrated and extendable desktop software platform for the organization and analysis of sequence data. Bioinformatics 28:1647-1649.

Kimura M (1980) A simple model for estimating evolutionary rates of base substitutions through comparative studies of nucleotide sequences. J Mol Evol 16: 111–120.

Kumar S, Stecher G, Li M, Knyaz C, Tamura K (2018) MEGA X: Molecular Evolutionary Genetics Analysis across computing platforms. Molecular Biology and Evolution 35:1547-1549

Nabhan AR, Sarkar IN (2012) The impact of taxon sampling on phylogenetic inference: a review of two decades of controversy. Briefings in bioinformatics 13:122-134.

**Table S1** List of the 26 cuticle hydrocarbons compounds (CHCs) found on *A. müelleri* workers, the correspondent hydrocarbon classes and the relative proportions (mean concentration and mean percentage). ID: identity; KI: Kovats’ index

| Hydrocarbon class | Compounds | ID | KI | Mean concentration | Mean % |
| --- | --- | --- | --- | --- | --- |
| Linear alkanes | *henicosane* | C_21_ | 2100 | 3.983 | 1.0 |
|  | *tricosane* | C_23_ | 2300 | 2.003 | 0.5 |
|  | *tetracosane* | C_24_ | 2400 | 6.079 | 1.5 |
|  | *pentacosane* | C_25_ | 2500 | 1.361 | 0.3 |
|  | *heptacosane* | C_27_ | 2700 | 25.642 | 6.3 |
|  | *octacosane* | C_28_ | 2800 | 2.141 | 0.5 |
|  | *nonacosane* | C_29_ | 2900 | 1.283 | 0.3 |
|  | *triacontane* | C_31_ | 3100 | 85.235 | 20.9 |
| Linear alkenes | *(Z)-nonadec-9-ene* | 9*Z*- C_19_ | 1867 | 8.951 | 2.2 |
|  | *(Z)-tricos-9-ene* | 9Z-C_23_ | 2263 | 31.078 | 7.6 |
|  | *(Z)-tricos-7-ene* | 7*Z*-C_23_ | 2274 | 1.349 | 0.3 |
|  | *(Z)-tetracos-9-ene* | 9*Z*-C_24_ | 2376 | 7.507 | 1.8 |
|  | *(Z)-pentacos-9-ene* | 9*Z*-C_25_ | 2470 | 127.540 | 31.3 |
|  | *(Z)-pentacos-7-ene* | 7*Z-*C_25_ | 2482 | 14.961 | 3.7 |
|  | *(Z)-hexacos-9-ene* | 9*Z*- C_26_ | 2568 | 3.832 | 0.9 |
|  | *(Z)-heptacos-9-ene* | 9*Z*-C_27_ | 2681 | 2.212 | 0.5 |
|  | *(Z)-octacos-9-ene* | 9*Z-*C_28_ | 2775 | 0.846 | 0.2 |
|  | *(E)-octacos-9-ene* | 9*E*-C_28_ | 2786 | 0.732 | 0.2 |
|  | *(Z)-nonacos-9-ene* | 9*Z*-C_29_ | 2879 | 47.621 | 11.7 |
|  | *(Z)-nonacos-7-ene* | 7*E*-C_29_ | 2888 | 4.048 | 1.0 |
|  | *(Z)-triacont-9-ene* | 9*Z*-C_31_ | 3078 | 11.759 | 2.9 |
|  | *(Z)-triacont-7-ene* | 7*Z*-C_31_ | 3085 | 5.666 | 1.4 |
| Methyl-branched alkanes | *13-methylpentacosane* | 13-Me-C_25_ | 2531 | 6.184 | 1.5 |
|  | *11-methylheptacosane* | 11-Me-C_27_ | 2728 | 0.694 | 0.2 |
|  | *11-methylnonacosane* | 11-Me-C_29_ | 2926 | 2.141 | 0.5 |
|  | *13-methyltriacontane* | 13-Me-C_31_ | 3130 | 3.116 | 0.8 |

**Fig S1** Neighbor-joining tree of COI sequence divergences (K2P) for all 52 individuals used in this study. Species names, Sample ID and Genbank numbers are given at branch tips. Only support values above 50% are shown


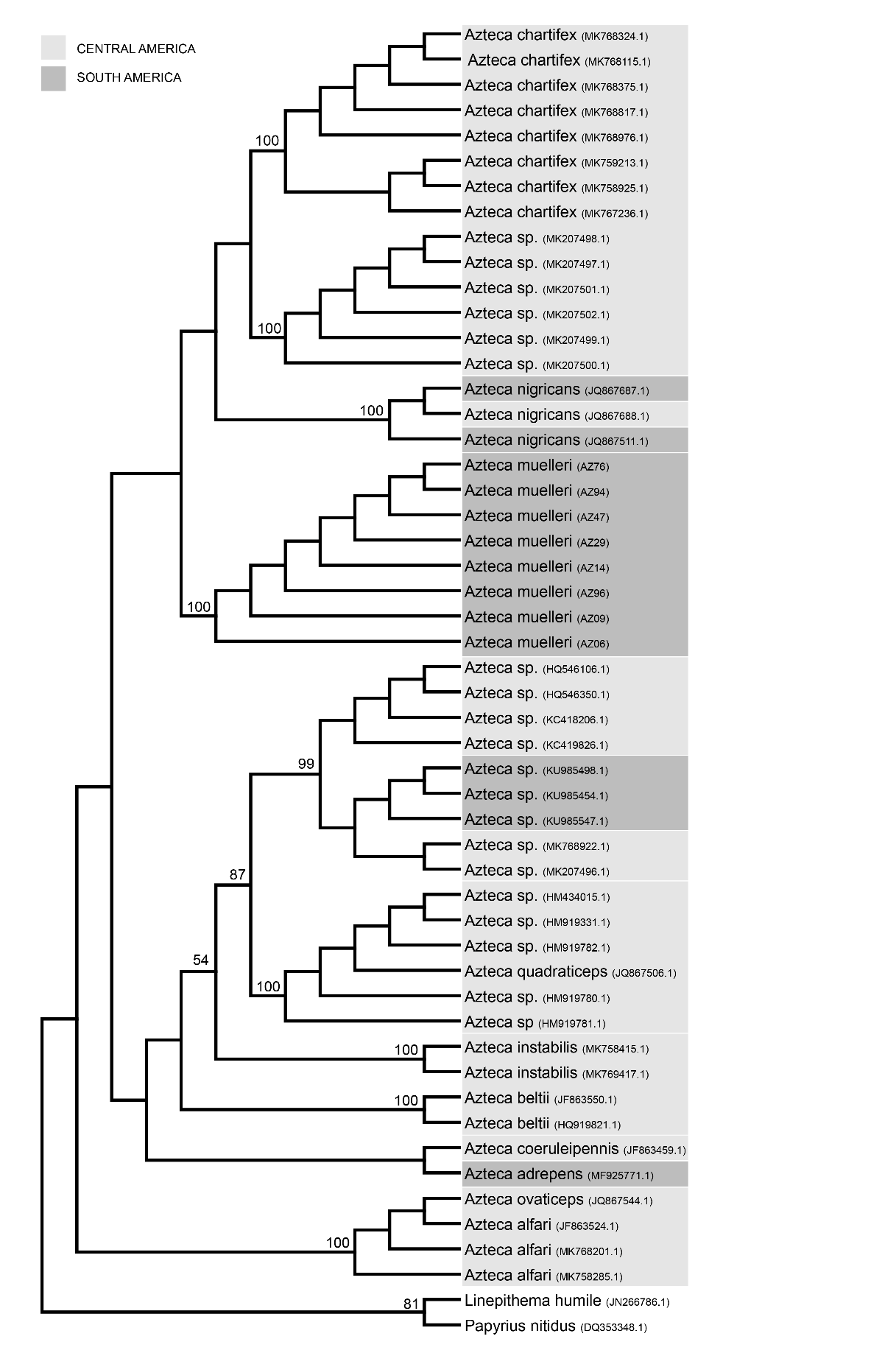

Supplement: Supplementary file 1 — Supplementary information. [file 41598_2021_85070_MOESM1_ESM.docx]
